# Salt Infusion in Morphia Poisoning

**Published:** 1902-05-17

**Authors:** 


### SALT INFUSION IN MORPHIA POISONING.

A case of poisoning by the subcutaneous injection of a large quantity of morphia (8 grains of the sulphate in three injections) is related by Dr. Edward F. Willoughby.[1] The case is interesting, in that the patient recovered even after such an enormous dose; but it is important, in view of the treatment adopted, namely, the subcutaneous infusion of normal saline solution. The patient had already had three injections of atropine and strychnine, and at least a dozen of ether. She was absolutely unconscious, with pin-point pupils, dusky complexion, and narcosis so complete that the application of the strongest possible faradic current to the soles of both feet did not excite the slightest movement even of the toes. A litre of saline solution was then infused by gravitation into the subcutaneous tissue of the flank. The effect was striking and almost immediate. Within 20 minutes the duskiness and coldness of the skin had given place to a natural colour and warmth, and the patient had so far recovered sensibility as to struggle violently against the mechanical and electrical stimulation which was being employed to keep her awake. She answered questions, but begged to be let alone. In addition to this treatment a weak solution of potassium permanganate was administered by the mouth with the object of breaking up any morphia which might be excreted into the intestinal canal. The next day the patient's condition was quite normal but for the sense of fatigue and soreness consequent on a score of hypodermic punctures and four hours of sustained artificial respiration. A very good case, and one well worth bearing in mind.

[1] Lancet, May 10.

